# Sit-To-Stand Movement Evaluated Using an Inertial Measurement Unit Embedded in Smart Glasses—A Validation Study

**DOI:** 10.3390/s20185019

**Published:** 2020-09-04

**Authors:** Justine Hellec, Frédéric Chorin, Andrea Castagnetti, Serge S. Colson

**Affiliations:** 1 Université Côte d’Azur, LAMHESS, EUR HEALTHY, 06205 Nice, France; justine.hellec@univ-cotedazur.fr (J.H.); chorin.f@chu-nice.fr (F.C.); 2 Ellcie Healthy, 06270 Villeneuve-Loubet, France; andrea.castagnetti@ellcie-healthy.com; 3 Université Côte d’Azur, CHU, Cimiez, Plateforme fragilité, 06000 Nice, France

**Keywords:** accelerometer, wearable sensors, vertical acceleration, reliability, chair rise test

## Abstract

Wearable sensors have recently been used to evaluate biomechanical parameters of everyday movements, but few have been located at the head level. This study investigated the relative and absolute reliability (intra- and inter-session) and concurrent validity of an inertial measurement unit (IMU) embedded in smart eyeglasses during sit-to-stand (STS) movements for the measurement of maximal acceleration of the head. Reliability and concurrent validity were investigated in nineteen young and healthy participants by comparing the acceleration values of the glasses’ IMU to an optoelectronic system. Sit-to-stand movements were performed in laboratory conditions using standardized tests. Participants wore the smart glasses and completed two testing sessions with STS movements performed at two speeds (slow and comfortable) under two different conditions (with and without a cervical collar). Both the vertical and anteroposterior acceleration values were collected and analyzed. The use of the cervical collar did not significantly influence the results obtained. The relative reliability intra- and inter-session was good to excellent (i.e., intraclass correlation coefficients were between 0.78 and 0.91) and excellent absolute reliability (i.e., standard error of the measurement lower than 10% of the average test or retest value) was observed for the glasses, especially for the vertical axis. Whatever the testing sessions in all conditions, significant correlations (*p* < 0.001) were found for the acceleration values recorded either in the vertical axis and in the anteroposterior axis between the glasses and the optoelectronic system. Concurrent validity between the glasses and the optoelectronic system was observed. Our observations indicate that the IMU embedded in smart glasses is accurate to measure vertical acceleration during STS movements. Further studies should investigate the use of these smart glasses to assess the STS movement in unstandardized settings (i.e., clinical and/or home) and to report vertical acceleration values in an elderly population of fallers and non-fallers.

## 1. Introduction

Sit-to-stand (STS) is a repeated movement of daily life with an average of 45 movements performed per day by adults [1]. This movement, requiring important muscular capacities [2], coordination and balance control is the prerequisite for all activities such as standing or walking [3]. For this reason, the STS movement, and its various forms of assessment (i.e., the duration to perform five [4] or ten [5] consecutive STS movements or the maximum number of STS movements performed in thirty seconds [6] or one minute [7]) is widely used to assess functional capacities of different populations in clinical settings [8,9]. In the literature, although the definition of the STS movement varies according to the aim of the study, many factors (e.g., chair seat height, speed, position of feet, use of armrests or not, etc.) influence STS performance [10]. Consequently, many studies have attempted to gain insight into the STS movement through biomechanical analyses with various systems such as force plates, combined with or without optoelectronic systems [8,11,12,13,14,15], video analysis [16], goniometry [17,18], and more recently accelerometry [15,19,20,21]. The recent technological progress involving wearable sensors allows for a more comprehensive analysis of the biomechanical kinematic variables of STS in clinical settings [22], which could be used in place of expensive and time-consuming biomechanical laboratory evaluations [23]. In addition, this kind of technology can be easily used in everyday life and could provide useful information in various clinical settings to monitor STS activity.

Due to their small size and portability, triaxial accelerometers were the first devices to be used to monitor physical activity and/or everyday movements in different settings [8,24,25,26]. More recently, some studies have investigated the relevance of using smartphones with incorporated accelerometers in clinical settings [27] and more particularly for the assessment of the STS [2,28,29,30,31]. Many studies have therefore attempted to evaluate STS movements with either a single accelerometer or multiple accelerometers, both embedded and not embedded in smartphones, placed over different regions of the human body. For example, a single accelerometer was placed over the chest [32], at the lower lumbar back level [2,8,19,30] or at the hip level [15,21,24], or multiple sensors were positioned over the trunk [20]. To date, there is no consensus on the optimal position to place the sensors for analysis of STS and other movements, and the optimal position might depend on the purpose of the movement being investigated. For example, it has been reported that movement regularity during walking and running was highest with a sensor located around the seventh cervical vertebra in comparison to a pelvic placement [33]. Linderman et al., [26] have also suggested that a sensor located at the head level could provide better kinematic information of the risk of falls in comparison to sensors located at the hip and the trunk. Nevertheless, a recent study demonstrated that a sensor strapped around the waist region provided better sensitivity in fall detection than one strapped over the head [34]. The authors expected to obtain higher accuracy with the sensor over the head and argued that during voluntary simulated falls, the participants may stabilize their head more than during unintended falls. Consequently, the kinematic analysis of daily life movements with an accelerometer located at the head level remains poorly investigated.

In this context, the use of an accelerometer embedded in glasses could be a promising and innovative technology to assess everyday life movements and/or could be useful for health-related applications. For example, it is estimated, and likely underestimated, that more than sixty percent of the population worldwide wears glasses [35,36]. As people get older, the use of vision correction glasses increases as well, with more than sixty percent of individuals aged between 40 and 50 years wearing glasses and this percentage growing up to ninety percent for individuals aged 75 or more. Consequently, for a great part of the population worldwide, glasses are a part of everyday life and are a wearable object and many other types of glasses are used to protect eyes from the sun and/or during the practice of various physical activities. Including sensors in such common objects that are part of everyday life would allow clinicians and researchers to collect remotely various biomechanical data during walking, running or even during repetitive movements, such as the STS. Considering that: (i) the STS movement is a recurrent activity of daily life and, (ii) STS performance can be used as an indicator for detecting the onset of the decline in physical abilities in the elderly, the present study aimed at investigating the acceleration during an STS movement with an everyday life wearable sensor such as glasses. In fact, to the best of our knowledge, no study has yet measured acceleration during an STS movement with an accelerometer embedded in glasses.

Knowing this, the purpose of this study was to determine the intra-session and inter-session reliability of smart glasses incorporating a triaxial accelerometer during the STS movement and to evaluate its concurrent validity against optoelectronic measurements in healthy participants in laboratory conditions.

## 2. Materials and Methods

### 2.1. Participants

Nineteen healthy adults without any known functional or cognitive impairments volunteered to participate in the study (9 men and 10 women; age: 26.4 ± 4.5 years old, height 172.7 ± 7.3 cm, body mass: 67.2 ± 14.3 kg, BMI: 22.4 ± 3.5 kg/m^2^; values are means and standard deviations). To be included, participants had to be able to stand up from a chair without the use of the arms, aged between 20 and 35 years, and without optical correction. The study was reviewed and approved by the South Mediterranean Protection of Persons Ethics Committee (registration number: 2015-A01188-41) and was conducted according to the Declaration of Helsinki revised in 2013. All participants were informed about the experimental procedures and informed written consent was provided prior to the study.

### 2.2. Study Design

This single-group repeated-measures study was conducted in a biomechanics laboratory (Frailty Platform) of the University Hospital Center in Nice (France). Participants attended the laboratory for two separate identical testing sessions, separated by one week. Each testing session was composed of STS movements performed in two speed conditions (i.e., comfort and slow). In addition, each speed was performed with and without the use of a cervical collar. For each experimental condition, fifteen repetitions were performed. Hence, the participants performed a total of 60 STS movements during each testing session. Participants were instructed to perform the STS from a starting position seated on a standardized chair (45 cm height from the floor) without armrests, arms folded across the chest without any obvious compensatory movement and keeping their feet on the floor during the test. The experimenter made sure that the instructions were fully understood by allowing practice trials before starting the testing session. During all the testing sessions, the participants wore the glasses without any optical correction.

### 2.3. Apparatus, Data Collection and Analysis

**Figure 1:**
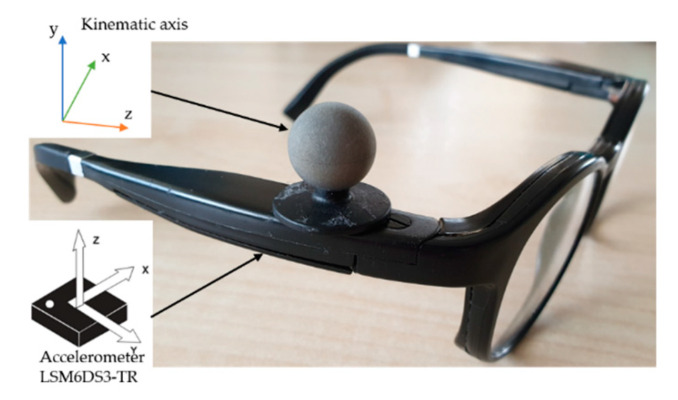
Position of the reflective marker on the right arm of the glasses and axes of the accelerometer and reflective marker.

Testing was carried out in a laboratory equipped with a Bertec instrumented treadmill (Bertec Corporation, Columbus, OH, USA) and six optoelectronic cameras (OptiTrack system, Naturalpoint, Inc., Corvallis, OR, USA). The portable inertial measurement unit (IMU) was embedded into the right arm of the smart glasses (Ellcie-Healthy, Villeneuve-Loubet, France). The IMU system is a microelectromechanical system (MEMS) LSM6DS3-TR (2.5 × 3 × 0.83 mm) [23]. This sensor is equipped with 3D-accelerometer and 3D-gyroscope. The orientation of the axes of the accelerometer embedded in the glasses is represented in Figure 1. The vertical axis of the glasses accelerometer was aligned with the gravitational axis while the participant was standing or sitting. In both positions, the accelerometer of the IMU recorded the gravitational acceleration (g) in the vertical axis. The range of measure of the IMU was ±2 g. A reflective kinematic marker was positioned on the right arm of the glasses in order to be as close as possible to the IMU. The orientation of the axes of the glasses kinematic marker is also represented in Figure 1. Another kinematic marker was positioned on the right-hand first finger of the participant. This marker was used to implement a synchronization procedure between both devices that acquire data simultaneously. The synchronization procedure involves the participant hitting the right branch of the glasses with his/her first finger. With a kinematic marker on the participant’s finger and by measuring the distance between the marker on the finger and the marker that was placed on the right branch of the glasses, it was possible to create a specific and easily detectable pattern in the optoelectronic signals. A similar pattern appeared in the acceleration measured by the IMU of the glasses, due to the effect of tapping with the finger near the accelerometer. During the offline synchronization procedure, the position in time of these two patterns was detected (i.e., one for the IMU signal of the glasses and the other one in the optoelectronic signal). This procedure allows us to determine the exact timestamp of the two events. Time alignment between the two signals was achieved by synchronizing these two events for all analyses.

**Figure 2:**
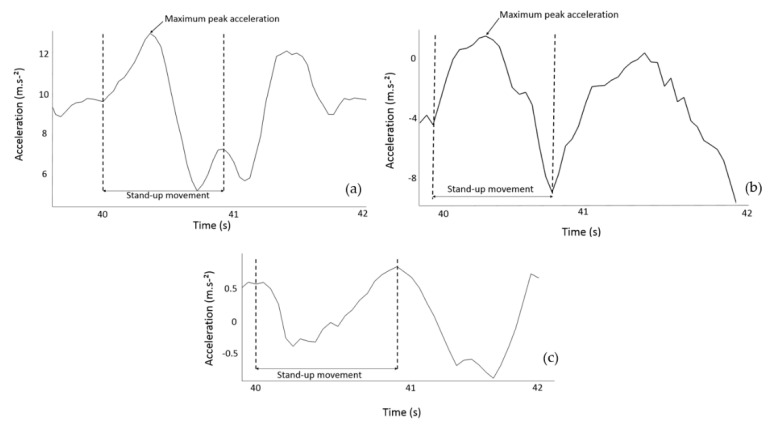
Acceleration signals from the glasses during a sit-to-stand movement in comfort speed without cervical collar on the three different axes: (**a**) vertical, (**b**) anteroposterior, and (**c**) mediolateral. Graphs a and b show the maximum acceleration peaks during the stand-up movement.

Raw IMU data recorded by the glasses were streamed via Bluetooth to a smartphone application (Driver by Ellcie-Healthy, Villeneuve-Loubet, France, Version 8.8) while kinematic data were recorded with the OptiTrack Motive software (Version 1.10). All data were analyzed using a Matlab program (The Mathworks, INC.; Natick, MA, USA, version R2018a). The raw acceleration of the IMU was directly provided by the accelerometer set at a sampling frequency of 26 Hz. Since the signal was recorded at a low frequency, filtering was not required. The maximum peak acceleration could be clearly identified in the raw signal with the synchronization procedure described previously. Moreover, during an STS movement there is no impact of the feet with the ground, such as during walking, which implies that few artifacts could influence the STS accelerometer signal. The acceleration signal was investigated during the transition from the initial seated position to the final standing position of the movement. The maximal acceleration values on both the vertical and anteroposterior axes were retained for the analysis (Figure 2). For the OptiTrack system, the position of the kinematic marker placed on the right branch of the glasses was derived twice to first compute the velocity of this marker and second, the acceleration. A moving average filter with a window length of five samples was applied to the derived signal to filter out noise and spurious events that can be generated by the derivation computation.

### 2.4. Statistical Analysis

Statistical analysis was performed with Statistica software (StatSoft, version 8.0, Tulsa, OK, USA). A three-layered approach was used in this study [37]. The Shapiro–Wilk test confirmed the normality of the data distribution. For each variable, a two-factor ANOVA with repeated measures (i.e., with or without cervical collar × intra- and inter-session) was performed to assess the systematic error within the fifteen trials and across both testing sessions. When necessary Bonferroni post-hoc tests were used to identify specific mean differences. The significance level was set to *p* < 0.05 and the effect size was computed from partial eta-squared values (η^2^*p*). The relative reliability of each variable across the trials within each testing session was assessed using intraclass correlation coefficients (ICC) with a 95% confidence interval. Test-retest reliability between both testing sessions was computed from the average values of the fifteen trials recorded during each session. ICC values less than 0.5, between 0.5 and 0.75, between 0.75 and 0.90 and greater than 0.90 were considered to have bad, moderate, good and excellent reliability, respectively [38]. The variability of each variable was assessed with the coefficient of variation (CV) to give information on the data stability between individuals rather than between trials [39]. The acceptability threshold was set at 10% [40]. The standard error of measurement (SEM), which is unaffected by inter-subject variability, was considered as an absolute reliability index [37]. SEM values smaller than 10% of the average test or retest value were considered to indicate excellent absolute reliability and minimal detectable change values with a confidence level of 95% (MDC95) were provided. Concurrent validity was first examined with the Pearson correlation coefficient (r). Then, Bland–Altman graphs with 95% limits of agreement were plotted to compare the maximal acceleration values of both systems [41].

## 3. Results

### 3.1. Reproducibility Intra- and Inter-Session of the Glasses and the Optoelectronic System

Importantly, no significant effect of the cervical collar was noted whatever the testing session and/or speed condition. Mean acceleration values of the IMU embedded in the glasses and the optoelectronic system are presented in Table 1 for both axes and for different experimental conditions.

For the acceleration recorded in the vertical axis, a significant interaction was found for the comfort speed measured with the glasses; F (14,504) = 1.82, *p* < 0.05, η^2^*p* = 0.05). Post-hoc analyses revealed that the first trial of the condition with the cervical collar was smaller than the other fourteen trials (0.001 < *p* < 0.05) for both sessions. No significant difference was observed for the slow speed for the glasses (*p* > 0.05). No significant differences were noted for either the comfort or the slow speed for the acceleration values measured with the optoelectronic system (*p* > 0.05).

In the anteroposterior axis, a significant interaction (i.e., within × between sessions) was found for the slow speed with the glasses; F (14,504) = 1.90, *p* < 0.05, η^2^*p* = 0.05. Pooled acceleration values of the first trial were smaller than the other fourteen trials, as well as some other trials between them (0.001 < *p* < 0.05) for both the condition with and without the cervical collar. A significant time effect (i.e., intra-session) was also observed for the comfort speed with the glasses [F (14,504) = 15.99, *p* < 0.001, η^2^*p* = 0.31] as well as for both the comfort [F (14,504) = 5.27, *p* < 0.001, η^2^*p* = 0.36] and slow [F (14,504) = 5.03, *p* < 0.001, η^2^*p* = 0.12] speed for the optoelectronic system. Mainly, pooled acceleration values of the first trial were smaller than the other trials and occasionally some trials between them (0.001 < *p* < 0.05).

### 3.2. Reliability Intra- and Inter-Session of the Glasses and the Optoelectronic System

The intra-session values of the ICC, SEM, MDC95 and CV of the accelerometer embedded in the glasses and the optoelectronic system are presented in Table 1 for both the vertical and the anteroposterior axes. For the glasses, all ICC values were between 0.78 and 0.91 indicating good to excellent reliability for the vertical axis. In the anteroposterior axis, values ranged from 0.70 to 0.89, indicating moderate to good reliability. The kinematic analysis of the optoelectronic system provided ICC values ranging from 0.84 to 0.94 and from 0.78 to 0.90 for the vertical and the anteroposterior axes, respectively.

SEM values of both systems were within the same range of values. Excellent absolute reliability (i.e., SEM smaller than 10% of the average test or retest value) was observed for the acceleration values of the vertical axis recorded with the glasses for all testing conditions (Table 1). Excellent absolute reliability was also observed for the optoelectronic system for the different conditions (Table 1).

The CV values of the acceleration measured with the glasses in the vertical axis were found to be of relatively small amplitude (i.e., less than 6.12%) whereas the CV values of the anteroposterior axis were between 37.61% and 64.35%. The CV values of the optoelectronic system ranged between 11.99% and 24.14% for both axes.

### 3.3. Concurrent Validity of the Glasses against the Optoelectronic System

**Figure 3:**
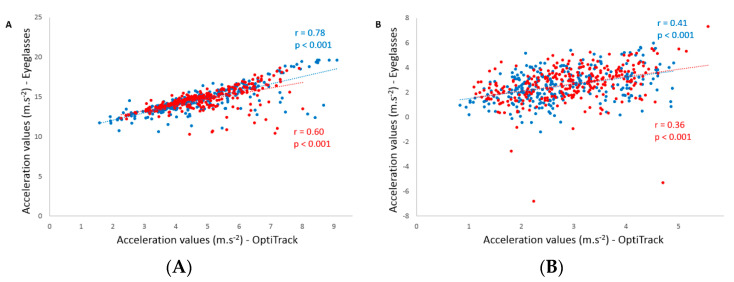
Pearson’s correlation of the acceleration values measured for the vertical (panel **A**) and anteroposterior (panel **B**) axes during comfort speed without the cervical collar for the first (blue dots) and the second (red dots) session.

**Figure 4:**
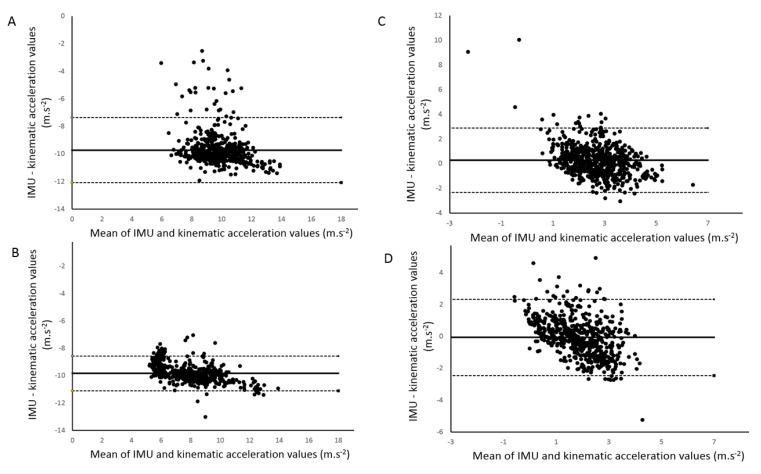
Bland–Altman plots for the acceleration values recorded without the cervical collar for the vertical axis (panel **A**, comfort speed; panel **B**, slow speed) and for the anteroposterior axis (panel **C**, comfort speed; panel **D**, slow speed), showing the differences between the two systems (i.e., inertial measurement unit (IMU) embedded in the glasses and kinematic analysis of the OptiTrack) against the mean of the two systems. The solid horizontal lines represent the estimated bias and the dashed lines represent the 95% limits of agreement.

Concurrent validity between the acceleration values of the IMU embedded in the glasses and the acceleration values computed from the kinematic analysis are displayed in Table 2. Significant Pearson correlations were found for both testing sessions in all conditions (*p* < 0.001). The coefficients ranged between 0.37 and 0.94 for the vertical axis and were between 0.36 and 0.61 for the anteroposterior axis. Figure 3 depicts an example of the correlations observed for the vertical and anteroposterior axes during comfort speed without the cervical collar. Bland–Altman plots were drawn for each condition and Figure 4 shows all the conditions performed without the cervical collar.

## 4. Discussion

To our knowledge, this is the first study to validate the use of an accelerometer embedded in smart glasses for measuring acceleration during an STS movement. Reliability and concurrent validity of acceleration values recorded with the glasses were evaluated for the vertical and anteroposterior axes for two different movement speeds (i.e., comfort and slow) and two conditions of head stabilization (i.e., with or without the cervical collar). Relative reliability intra- and inter-session was good to excellent, especially for the vertical axis. In addition, excellent absolute reliability was observed for the glasses in the vertical axis. Finally, concurrent validity of the glasses against the kinematic data obtained from a motion capture system was demonstrated.

In a sample population of healthy adults, vertical acceleration values, recorded with the motion capture system from the reflective marker located on the arm of the glasses, are consistent with acceleration values of the center of mass during the STS movement reported in previous studies [20]. Similarly, the acceleration values obtained with our glasses agree with those recorded with a motion sensor located at the hip in young healthy participants during an STS movement [21]. Importantly, the use of a cervical collar did not significantly influence acceleration values, suggesting that assessing the STS movement without head stabilization is feasible. In addition, during the comfort speed with the cervical collar, we observed that the acceleration value of the first trial was smaller than the other trials for both sessions. This observation was also true for the first trial during comfort speed without the cervical collar, but only with respect to the third and the fifth trials. Taken together, these observations suggest that even if the participants were familiarized with the movement, they may still need at least one trial to perform correctly when the testing session starts. In fact, it was recently reported that a minimum of two trials were required to obtain excellent test-retest reliability of the acceleration during normal speed STS in young adults [21]. Nonetheless, the relative reliability of the glasses across the fifteen trials within each testing session was excellent for almost all the conditions (i.e., 0.86 < ICC < 0.91; Table 1). Only the second testing session for the comfort speed with cervical collar condition showed a smaller ICC value of 0.78. These observations, combined with a coefficient of variation less than 7%, indicate that the measurement of the vertical acceleration with the glasses is highly reliable and reproducible. Moreover, we also observed that the SEM for all testing conditions and sessions was smaller than 10%, indicating excellent absolute reliability [37]. The relative reliability of the glasses was similar to the acceleration values obtained from the kinematic analysis, even though the coefficient of variation was greater than 12% for the latter. In addition, with comparable SEM, the kinematic analysis had lower absolute reliability than the glasses. Consequently, the use of glasses to assess vertical acceleration during the STS movement is highly reliable.

Test-retest evaluation of the vertical acceleration across the two testing sessions shows good reliability for almost all testing conditions (i.e., ICC > 0.75; Table 1), since only the slow speed with the cervical collar exhibited a moderate ICC value of 0.63. The ICC values of the kinematic analysis were within the same range, with smaller values than the glasses. In addition, the SEM was less than 10%, indicating excellent absolute reliability for the glasses, which was not the case for the kinematic analysis. Our test-retest ICCs of the vertical acceleration values were comparable to or higher than values reported during STS at normal speed in young adults [21]. It is also worth mentioning that the test-retest ICC values obtained here are consistent with other studies assessing the kinematic variables or scores of the STS movement with different systems in young adults [18,42].

Concurrent validity between the glasses and the kinematic analysis was investigated for all conditions within and between testing sessions. Strong to very strong correlations were found between systems for the slow speed condition whereas weak to strong correlations were observed for the comfort speed within testing sessions (Table 2). Indeed, similar to the abovementioned ICC values, the second testing session for the comfort speed with the cervical collar condition showed a weak correlation. However, the most important observation was that the highest coefficients of correlation for each speed were systematically found for the condition without head stabilization, as is performed in everyday life (Figure 3). For these two conditions, test-retest measures exhibited strong to very strong correlations. Although more variable in the comfort speed condition, another interesting result is the higher vertical acceleration values measured with the eyeglasses compared to the acceleration obtained from the kinematic analysis. In fact, Bland–Altman analyses did not suggest a systematic bias in the measurements between systems when acceleration values are increasing. The only “bias” that could be observed is related to the uncorrected gravitational acceleration measured with the glasses compared to the kinematic analysis. Knowing this, we can conclude that both systems are comparable in the measurement of vertical acceleration.

In this study, the acceleration values measured in the anteroposterior axis were also investigated. Both the glasses and the kinematic analysis had moderate to good reliability within each testing session for all conditions. Although the reliability of the glasses for this axis is acceptable, the coefficients of variation are larger than the ones obtained from the kinematic analysis. Nevertheless, the kinematic analysis did not show excellent absolute reliability either, for this axis, and the coefficients of variation were greater than 16%. In addition, although they are within the same range of values, SEM values were greater than 10%, indicating that excellent absolute reliability was not obtained for this axis. ICC values of the acceleration for the test-retest evaluation were even worse for the glasses. Concurrent validity of the glasses against the kinematic analysis was moderate according to the coefficient of correlation. However, Bland–Altman plots emphasized that no bias was found between the systems and the limits of agreement were within the same range of values observed for the vertical acceleration. Taken together, these results suggest that caution should be taken when investigating acceleration values of the head in the anteroposterior axis during STS movements, especially with glasses.

It is extremely difficult to compare our results with previous studies since no study has directly investigated acceleration during an STS movement from an IMU located at the head level against a motion capture system in young healthy adults. However, a recent study highlighted that maximal vertical acceleration measured at the hip with a motion sensor in older adults had adequate accuracy and concurrent validity against a standard laboratory method in which vertical acceleration was determined from force plate’s measurements [14]. These authors have reported a strong association between the motion sensor and force plate methods for vertical hip acceleration. Although measurement systems and methods differ, we observed a similar level of association in the present study for vertical acceleration measured at the head. Consequently, investigating vertical acceleration during human movements, such as the STS with an IMU embedded in glasses, seems promising.

Some limitations of the study should be considered. The main methodological limitation concerns the absence of a calibration procedure between the global reference frame of the optoelectronic system and the local reference frame of the IMU embedded in the glasses. Although this procedure is important to accurately compare the acceleration values recorded by the two systems (i.e., to render the two measurements fully consistent and comparable), it was not performed in the present study since it does not reflect the “real” use of the glasses in daily life. Indeed, in ecological conditions each individual may wear the glasses differently (i.e., due to individual’s anatomical landmarks, the branches of the glasses might not be strictly perpendicular to the gravitational acceleration) and subtle head movements could also be realized during STS movements and/or any kind of daily life movement. Even if a possible bias could have influenced the results of the present investigation due to the absence of a calibration procedure, the best absolute reliability of the acceleration values recorded here was found for the glasses during STS movements performed without the cervical collar, hence reflecting daily life conditions. Additionally, this study did not investigate different sensor locations nor attempt to validate the vertical acceleration measured with the glasses against other biomechanical variables such as the vertical acceleration derived from force plate recordings. Since the measurements were done in a laboratory environment, it would now be interesting to assess the STS movement in unstandardized settings (i.e., clinical and/or at home) reflecting daily life conditions. Finally, although the STS movement is relevant to assess functional status in various clinical settings, only young healthy adults were enrolled in the study, so the reliability and the accuracy validity of the glasses should be investigated in the elderly and health-compromised populations.

## 5. Conclusions

The investigation of everyday movements with inertial sensors has recently gained in popularity. STS is widely used to assess functional status in different populations. The objective of this study was to validate the use of an IMU embedded in glasses for the evaluation of the STS movement. The IMU embedded in glasses is reproducible and valid for vertical acceleration measurements. Knowing that this movement is automatized in most individuals, its use in everyday life is promising as well as in clinical settings. Indeed, with a simple everyday object, it is therefore possible to analyze the STS movement instantaneously in a clinical setting or gait laboratory without time-consuming signal processing and/or remotely at home through the smartphone application associated with the glasses. In an ecological situation, with such continuous remote recording of STS movements of daily life, it could be possible to detect a potential decline in physical capacities and, for example, prevent the risk of falls in the elderly. Future work should determine vertical acceleration values obtained with the glasses in an elderly population of fallers and non-fallers.

## Figures and Tables

**Table 1:** Mean acceleration values, intraclass correlation coefficient (95% confidence interval), standard error of the measurement, minimum detectable change and coefficient of variation (%) measured with the eyeglasses and the kinematic system (OptiTrack) during fifteen sit-to-stand movements for both sessions.

	Mean Acceleration Value (Standard Error) in m·s^−2^		Intraclass Correlation Coefficient (95% Confidence Interval)			Standard Error of the Measurement			Minimum Detectable Change			Coefficient of Variation (%)	
	Session 1	Session 2	Session 1	Session 2	Test-Retest	Session 1	Session 2	Test-Retest	Session 1	Session 2	Test-Retest	Session 1	Session 2
**Glasses**													
**VA**													
Comfort WCC	14.65 (1.65)	14.63 (1.19)	0.88 (0.79–0.94)	0.86 (0.76–0.93)	0.87 (0.69–0.95)	**0.60**	**0.53**	**0.52**	1.65	1.46	1.44	5.17	5.33
Comfort CC	13.29 (1.70)	13.49 (1.04)	0.90 (0.82–0.95)	0.78 (0.66–0.89)	0.83 (0.61–0.93)	**0.59**	**0.61**	**0.60**	1.63	1.70	1.66	6.12	5.79
Slow WCC	13.13 (1.74)	12.57 (1.72)	0.89 (0.81–0.95)	0.91 (0.85–0.96)	0.75 (0.46–0.90)	**0.61**	**0.54**	**0.87**	1.68	1.49	2.41	4.71	4.63
Slow CC	11.94 (1.68)	11.71 (1.35)	0.91 (0.84–0.96)	0.90 (0.83–0.95)	0.63 (0.25–0.84)	**0.53**	**0.46**	**0.92**	1.47	1.27	2.56	4.84	4.65
**APA**													
Comfort WCC	2.50 (0.99)	2.65 (0.94)	0.72 (0.58–0.85)	0.70 (0.55–0.84)	0.45 (0.01–0.74)	0.47	0.55	0.73	1.24	1.51	2.01	37.61	53.32
Comfort CC	1.76 (0.85)	2.12 (1.21)	0.79 (0.66–0.89)	0.88 (0.79–0.94)	0.51 (0.08–0.78)	0.36	0.75	0.76	1.01	2.08	2.11	59.84	33.06
Slow WCC	2.01 (0.99)	1.80 (1.19)	0.80 (0.69–0.90)	0.82 (0.71–0.91)	0.60 (0.21–0.82)	0.42	0.42	0.70	1.17	1.16	1.93	53.45	56.88
Slow CC	1.24 (1.12)	1.20 (1.28)	0.78 (0.66–0.89)	0.89 (0.81–0.95)	0.67 (0.32–0.86)	0.40	0.48	0.70	1.11	1.32	1.94	33.32	64.35
**OptiTrack**													
**VA**													
Comfort WCC	4.85 (1.46)	4.95 (1.04)	0.90 (0.83–0.95)	0.84 (0.74–0.92)	0.92 (0.81–0.97)	0.50	**0.47**	0.36	1.38	1.30	1.00	13.77	11.99
Comfort CC	4.95 (1.64)	5.31 (1.42)	0.92 (0.86–0.96)	0.87 (0.79–0.94)	0.72 (0.40–0.88)	**0.49**	0.59	0.81	1.37	1.55	2.26	18.53	17.67
Slow WCC	3.27 (1.34)	2.74 (1.47)	0.87 (0.78–0.94)	0.94 (0.89–0.97)	0.72 (0.40–0.88)	0.51	0.37	0.76	1.42	1.03	2.10	19.31	19.18
Slow CC	3.14 (1.31)	2.88 (1.61)	0.84 (0.74–0.92)	0.92 (0.86–0.96)	0.59 (0.21–0.82)	0.57	0.47	0.93	1.57	1.31	2.57	19.11	21.91
**APA**													
Comfort WCC	2.77 (0.80)	2.93 (0.77)	0.79 (0.68–0.90)	0.80 (0.68–0.90)	0.94 (0.85–0.98)	0.43	0.41	**0.20**	1.18	1.14	0.55	17.67	16.26
Comfort CC	2.85 (0.68)	3.01 (0.61)	0.79 (0.66–0.89)	0.78 (0.66–0.89)	0.70 (0.37–0.87)	0.38	0.38	0.37	1.05	1.04	1.02	17.88	18.05
Slow WCC	1.93 (0.75)	1.73 (0.76)	0.83 (0.73–0.92)	0.90 (0.82–0.95)	0.74 (0.45–0.89)	0.36	0.27	0.39	0.99	0.74	1.09	23.54	22.72
Slow CC	1.93 (0.78)	1.79 (0.78)	0.82 (0.71–0.92)	0.82 (0.71–0.91)	0.86 (0.68–0.95)	0.36	0.36	0.30	1.01	1.01	0.82	21.55	24.14

Values in bold indicate excellent absolute reliability (i.e., SEM value smaller than 10% of the average test or retest value). Abbreviations: WCC, without cervical collar; CC, with cervical collar; APA, anteroposterior axis; VA, vertical axis.

**Table 2:** Concurrent validity of the acceleration values obtained from the glasses compared to the OptiTrack.

	Pearson Correlation (r)			Bland-Altman		
	Session 1	Session 2	Global	Bias (Mean Difference)	Lower Limit	Upper Limit
VA						
Comfort WCC	0.78 ***	0.60 ***	0.72 ***	−9.73	−12.10	−7.35
Comfort CC	0.62 ***	0.37 ***	0.52 ***	−8.29	−11.30	−5.29
Slow WCC	0.94 ***	0.94 ***	0.94 ***	−9.84	−11.12	−8.56
Slow CC	0.76 ***	0.80 ***	0.76 ***	−8.84	−10.89	−6.79
APA						
Comfort WCC	0.41 ***	0.36 ***	0.38 ***	0.28	−2.33	2.88
Comfort CC	0.43 ***	0.61 ***	0.53 ***	0.97	−1.24	3.18
Slow WCC	0.42 ***	0.48 ***	0.46 ***	−0.07	−2.46	2.31
Slow CC	0.54 ***	0.43 ***	0.48 ***	0.64	−1.84	3.12

*** indicates significant correlation between systems (*p* < 0.001). Abbreviations: WCC, without cervical collar; CC, with cervical collar; APA, anteroposterior axis; VA, vertical axis.

## References

[1] Bohannon R.W. (2015). Daily Sit-to-Stands Performed by Adults: A Systematic Review. J. Phys. Ther. Sci..

[2] Cerrito A., Bichsel L., Radlinger L., Schmid S. (2015). Reliability and Validity of a Smartphone-Based Application for the Quantification of the Sit-to-Stand Movement in Healthy Seniors. Gait Posture.

[3] Janssen W.G.M., Bussmann J.B.J., Horemans H.L.D., Stam H.J. (2005). Analysis and Decomposition of Accelerometric Signals of Trunk and Thigh Obtained during the Sit-to-Stand Movement. Med. Biol. Eng. Comput..

[4] Guralnik J.M., Simonsick E.M., Ferrucci L., Glynn R.J., Berkman L.F., Blazer D.G., Scherr P.A., Wallace R.B. (1994). A Short Physical Performance Battery Assessing Lower Extremity Function: Association with Self-Reported Disability and Prediction of Mortality and Nursing Home Admission. J. Gerontol..

[5] Csuka M., McCarty D.J. (1985). Simple Method for Measurement of Lower Extremity Muscle Strength. Am. J. Med..

[6] Jones C.J., Rikli R.E., Beam W.C. (1999). A 30-s Chair-Stand Test as a Measure of Lower Body Strength in Community-Residing Older Adults. Res. Q. Exerc. Sport.

[7] Koufaki P., Mercer T.H., Naish P.F. (2002). Effects of Exercise Training on Aerobic and Functional Capacity of End-Stage Renal Disease Patients. Clin. Physiol. Funct. Imaging.

[8] Zijlstra A., Mancini M., Lindemann U., Chiari L., Zijlstra W. (2012). Sit-Stand and Stand-Sit Transitions in Older Adults and Patients with Parkinson’s Disease: Event Detection Based on Motion Sensors versus Force Plates. J. Neuroeng. Rehabil..

[9] Millington P.J., Myklebust B.M., Shambes G.M. (1992). Biomechanical Analysis of the Sit-to-Stand Motion in Elderly Persons. Arch. Phys. Med. Rehabil..

[10] Janssen W.G., Bussmann H.B., Stam H.J. (2002). Determinants of the Sit-to-Stand Movement: A Review. Phys. Ther..

[11] Kralj A., Jaeger R.J., Munih M. (1990). Analysis of Standing up and Sitting down in Humans: Definitions and Normative Data Presentation. J. Biomech..

[12] Pai Y.-C., Rogers M.W. (1991). Speed Variation and Resultant Joint Torques during Sit-to-Stand. Arch. Phys. Med. Rehabil..

[13] Chorin F., Cornu C., Beaune B., Frère J., Rahmani A. (2016). Sit to Stand in Elderly Fallers vs Non-Fallers: New Insights from Force Platform and Electromyography Data. Aging Clin. Exp. Res..

[14] Regterschot G.R.H., Zhang W., Baldus H., Stevens M., Zijlstra W. (2016). Accuracy and Concurrent Validity of a Sensor-Based Analysis of Sit-to-Stand Movements in Older Adults. Gait Posture.

[15] Hellmers S., Fudickar S., Lau S., Elgert L., Diekmann R., Bauer J., Hein A. (2019). Measurement of the Chair Rise Performance of Older People Based on Force Plates and IMUs. Sensors.

[16] Shukla B.K., Jain H., Vijay V., Yadav S.K., Mathur A., Hewson D.J. (2020). A Comparison of Four Approaches to Evaluate the Sit-to-Stand Movement. IEEE Trans. Neural Syst. Rehabil. Eng..

[17] Shepherd R.B., Gentile A.M. (1994). Sit-to-Stand: Functional Relationship between Upper Body and Lower Limb Segments. Hum. Mov. Sci..

[18] Pourahmadi M.R., Ebrahimi Takamjani I., Jaberzadeh S., Sarrafzadeh J., Sanjari M.A., Bagheri R., Jannati E. (2018). Test-Retest Reliability of Sit-to-Stand and Stand-to-Sit Analysis in People with and without Chronic Non-Specific Low Back Pain. Musculoskelet. Sci. Pract..

[19] Millor N., Lecumberri P., Gómez M., Martínez-Ramírez A., Izquierdo M. (2013). An Evaluation of the 30-s Chair Stand Test in Older Adults: Frailty Detection Based on Kinematic Parameters from a Single Inertial Unit. J. Neuroeng. Rehabil..

[20] Zijlstra W., Bisseling R.W., Schlumbohm S., Baldus H. (2010). A Body-Fixed-Sensor-Based Analysis of Power during Sit-to-Stand Movements. Gait Posture.

[21] Regterschot G.R.H., Zhang W., Baldus H., Stevens M., Zijlstra W. (2014). Test–Retest Reliability of Sensor-Based Sit-to-Stand Measures in Young and Older Adults. Gait Posture.

[22] Giansanti D., Maccioni G. (2006). Physiological Motion Monitoring: A Wearable Device and Adaptative Algorithm for Sit-to-Stand Timing Detection. Physiol. Meas..

[23] Janssen W.G.M., Bussmann J.B.J., Horemans H.L.D., Stam H.J. (2008). Validity of Accelerometry in Assessing the Duration of the Sit-to-Stand Movement. Med. Biol. Eng. Comput..

[24] Regterschot G.R.H., Folkersma M., Zhang W., Baldus H., Stevens M., Zijlstra W. (2014). Sensitivity of Sensor-Based Sit-to-Stand Peak Power to the Effects of Training Leg Strength, Leg Power and Balance in Older Adults. Gait Posture.

[25] Yang C.-C., Hsu Y.-L. (2010). A Review of Accelerometry-Based Wearable Motion Detectors for Physical Activity Monitoring. Sensors.

[26] Lindemann U., Hock A., Stuber M., Keck W., Becker C. (2005). Evaluation of a Fall Detector Based on Accelerometers: A Pilot Study. Med. Biol. Eng. Comput..

[27] Ozdalga E., Ozdalga A., Ahuja N. (2012). The Smartphone in Medicine: A Review of Current and Potential Use Among Physicians and Students. J. Med. Internet Res..

[28] Ruiz-Cárdenas J.D., Rodríguez-Juan J.J., Smart R.R., Jakobi J.M., Jones G.R. (2018). Validity and Reliability of an IPhone App to Assess Time, Velocity and Leg Power during a Sit-to-Stand Functional Performance Test. Gait Posture.

[29] Roldán Jiménez C., Bennett P., Ortiz García A., Cuesta Vargas A.I. (2019). Fatigue Detection during Sit-To-Stand Test Based on Surface Electromyography and Acceleration: A Case Study. Sensors.

[30] Galán-Mercant A., Cuesta-Vargas A.I. (2013). Differences in Trunk Accelerometry Between Frail and Nonfrail Elderly Persons in Sit-to-Stand and Stand-to-Sit Transitions Based on a Mobile Inertial Sensor. JMIR Mhealth Uhealth.

[31] Orange S.T., Metcalfe J.W., Liefeith A., Jordan A.R. (2020). Validity of Various Portable Devices to Measure Sit-to-Stand Velocity and Power in Older Adults. Gait Posture.

[32] Ganea R., Paraschiv-Ionescu A., Büla C., Rochat S., Aminian K. (2011). Multi-Parametric Evaluation of Sit-to-Stand and Stand-to-Sit Transitions in Elderly People. Med. Eng. Phys..

[33] Rabuffetti M., Scalera G., Ferrarin M. (2019). Effects of Gait Strategy and Speed on Regularity of Locomotion Assessed in Healthy Subjects Using a Multi-Sensor Method. Sensors.

[34] Özdemir A. (2016). An Analysis on Sensor Locations of the Human Body for Wearable Fall Detection Devices: Principles and Practice. Sensors.

[35] GlassesCrafter.com. http://www.glassescrafter.com/information/percentage-population-wears-glasses.html.

[36] Epsilon. https://www.epsilon.insee.fr/jspui/bitstream/1/23534/1/er881.pdf.

[37] Weir J.P. (2005). Quantifying Test-Retest Reliability Using the Intraclass Correlation Coefficient and the SEM. J. Strength Cond. Res..

[38] Koo T.K., Li M.Y. (2016). A Guideline of Selecting and Reporting Intraclass Correlation Coefficients for Reliability Research. J. Chiropr. Med..

[39] Knutson L.M., Soderberg G.L., Ballantyne B.T., Clarke W.R. (1994). A Study of Various Normalization Procedures for within Day Electromyographic Data. J. Electromyogr. Kinesiol..

[40] Atkinson G., Nevill A.M. (1998). Statistical Methods for Assessing Measurement Error (Reliability) in Variables Relevant to Sports Medicine. Sports Med..

[41] Bland J.M., Altman D.G. (1986). Statistical Methods for Assessing Agreement between Two Methods of Clinical Measurement. Lancet.

[42] Yamako G., Chosa E., Totoribe K., Fukao Y., Deng G. (2017). Quantification of the Sit-to-Stand Movement for Monitoring Age-Related Motor Deterioration Using the Nintendo Wii Balance Board. PLoS ONE.

